# Few-Shot Ensemble Learning for Catalysis and Application to Trimetallics for Oxygen Reduction

**DOI:** 10.1021/acscatal.5c08168

**Published:** 2026-03-02

**Authors:** Avery F. Hill, Andrea Ruiz-Escudero, Matthew M. Montemore

**Affiliations:** † Department of Chemical and Biomolecular Engineering, 5783 Tulane University, 6823 St. Charles Ave., New Orleans, Louisiana 70118, United States; ‡ Department of Computer Science and Information Technologies, Faculty of Computer Science, University of A Coruña, Campus de Elviña, A Coruña 15071, Galicia , Spain; § Department of Organic and Inorganic Chemistry, 16402 University of the Basque Country UPV/EHU, Barrio Sarriena s/n, Leioa 48940, Bizkaia , Spain

**Keywords:** machine-learned interatomic potentials, few-shot learning, bias correction, adsorption energy, trimetallic catalysts, oxygen reduction reaction

## Abstract

Machine-learned interatomic potentials (MLIPs) are increasingly used to accelerate catalyst discovery, but their accuracy and utility are often unclear, particularly when applying them to different computational setups or design spaces than those of the training data. This hinders their effective use in catalyst screening. Here, we improve accuracy and provide reliable uncertainty quantification through an ensemble-based, few-shot transfer learning strategy. The framework applies a bias-correction procedure to an ensemble of catalysis-focused MLIPs using a small number of density functional theory (DFT) labels from the target setup and design space. Applied to OH adsorption on bimetallic alloys, the approach reduces root mean squared errors (RMSEs) by 60% on average after incorporating just one additional DFT-calculated adsorption energy. For H adsorption on single-atom alloys, even the zero-shot ensemble is more accurate than any of the MLIPs, with further improvements when three to five DFT calculations are used for bias correction. The corrected ensemble yields well-calibrated uncertainty estimates, with miscalibration areas of just 0.039 and 0.088 for the two data sets. In a proof-of-concept screening campaign, the method identified a promising trimetallic candidate catalyst for the oxygen reduction reaction using only four total DFT-calculated adsorption energies. Taken together, these results demonstrate that few-shot bias correction enables reliable transfer of MLIP predictions across mismatched alloy search spaces and DFT methodologies, providing a practical route to accurate, uncertainty-aware catalyst screening with high efficiency.

## Graphical abstract

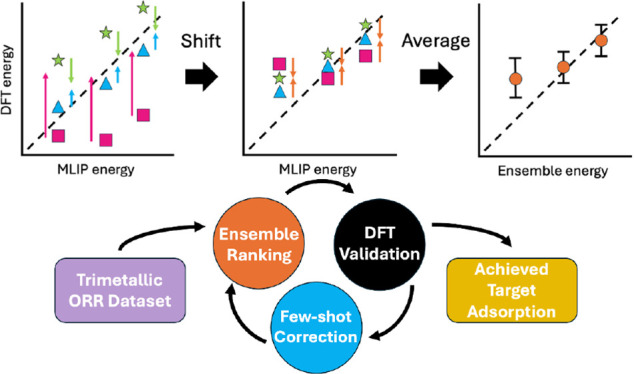

## Introduction

Quantum-based calculations can significantly accelerate catalyst discovery by identifying promising candidates prior to experimental synthesis.^1–3^ Nevertheless, the space of possible catalysts is combinatorially large, and realistic models often require large system sizes to include extended surfaces, solvent, or high adsorbate coverages. Thus, brute-force screening of either large design spaces or complex structures is computationally prohibitive for quantum methods even with modern supercomputing resources.^4^

For this reason, researchers are increasingly turning to machine learning (ML) models to approximate quantum methods at a much lower cost. In particular, ML interatomic potentials (MLIPs) are often trained on large databases to promote generalizability and can predict energies and forces in close agreement with traditional quantum calculations while requiring only a fraction of the computational effort.^5,6^ However, when using these models in practice for screening, issues can arise that degrade the ease and efficiency of use. First, a user may prefer a different computational setup than was used for the training data, for a multitude of possible reasons including consistency with the user’s past data and knowledge of an effective setup for the target design space. This can lead to systematic mismatches.^7,8^ Second, even when MLIPs have low average errors, large errors still occur in some cases, particularly when the screening space begins to diverge from the training data. From a ML perspective, it is important to consider these inconsistencies arising from a distribution shift between the training and target domains.^9,10^

To account for distribution shifts, researchers sometimes fine-tune the models with additional data, updating the MLIP parameters to improve performance,^9,11^ including through physics-constrained data-augmentation strategies that generate additional configurations to improve coverage of specific regions of configuration space.^12^ However, collecting the tens to thousands of reference calculations needed for fine-tuning can significantly slow down screening,^4,13^ particularly when using high-level calculations, studying large systems, or if considering many intermediates in a catalytic reaction. These challenges have motivated the development of alternative approaches that can adapt MLIPs with only a small number of new samples. Such few-shot methods aim to improve predictions with as few as zero to five reference calculations, making them especially attractive in data-scarce applications.^14,15^ As an example, one such strategy is test-time adaptation (TTA), a family of zero-shot methods. Originally developed in computer vision,^16^ TTA methods have recently been extended to materials science and MLIP applications.^10,17^ They allow pretrained models to self-correct during inference, improving performance in unfamiliar chemical environments. However, these zero-shot methods are not guaranteed to succeed: without access to labeled feedback, their self-consistency objectives can reinforce incorrect predictions.^18^ Since ML models in materials science usually cannot guarantee low errors across all materials in a design space, promising candidates should generally be validated with DFT. The resulting data is valuable and motivates few-shot approaches: by explicitly incorporating these small amounts of labeled data in an iterative search, such methods can correct systematic distribution biases between data sets while maintaining data efficiency. Related strategies such as active learning and Bayesian optimization also leverage iterative DFT calculations to guide exploration by selecting which new calculations to perform.^19–22^ However, these frameworks primarily focus on data acquisition and usually require repeated retraining or tight integration between model and search loop. In contrast, the present work assumes that DFT validation is already being performed and seeks to directly exploit these sparse labels to adapt predictions from pretrained MLIPs without retraining or access to training data.

Two recent studies have demonstrated few-shot MLIP adaptation in a materials context. Fine-tuning a foundation model using 1 to 20 perturbed supercell configurations derived from a single equilibrium LiBr structure enables efficient recovery of multiple properties within a fixed material,^23^ but is not designed to address adaptation to new compositions. Correcting MLIP force predictions by attaching a linear correction layer and fitting it using the many force components available from a single configuration of Li_2_B_3_PO_8_ represents an effective strategy for single-system force refinement.^9^ However, the reliance on force labels from one structure makes it less straightforward to extend to system-level energetic quantities, such as adsorption energies, where only one energy label is available per system for adaptation. Thus, very few studies have addressed the few-shot regime in a way that is useful for catalyst screening.

Alongside these single-system adaptation strategies, recent efforts that explicitly target system-level energetic prediction across materials have focused on improving data efficiency through transfer learning, meta-learning, and multitask frameworks, yet still require tens to hundreds of additional training samples. For example, past studies have used a transfer-learning framework that reuses pretrained graph representations via Fourier features to accelerate retraining,^24^ and meta-learning to improve cross-system generalization of foundation MLIPs.^25^ Other approaches, such as transfer learning using attentions across atomic graphs (TAAG), fine-tune only lightweight attention layers on top of frozen pretrained representations, thereby reducing the amount of target-domain data needed for adaptation across materials.^26^ Similarly, a mixture-of-experts approach has been used to combine models trained on diverse data sets, enhancing coverage under data scarcity.^27^ Rather than fine-tuning MLIPs directly, it is possible to create transferable frameworks for derived quantities like adsorption energies. For example, a latent-variable framework has been developed to improve transferability and interpretability of ML models for catalysis, enabling efficient adaptation across adsorbates and surfaces,^28^ and a more physically motivated ML model showed transferability across different types of alloys.^29^ Other studies have created relatively general models for adsorption.^30–32^ Collectively, these methods advance transferability and data efficiency, but primarily focus on adaptation within fixed systems or require substantially more training data than the few-shot regime targeted in this work. Furthermore, they typically require fairly specialized knowledge in transfer learning as well as access to the training data. Thus, a practical technique that can use existing, pretrained ML models and treat them as black-box methods would significantly improve the ease of use and the possibility of broad adoption.

Even when model transfer is successful, reliable deployment is greatly facilitated by estimating which predictions can be trusted. While simpler systematic biases may be corrected, MLIPs can still fail on individual systems.^33,34^ Uncertainty quantification provides a statistical framework for estimating model confidence in each prediction, allowing unreliable predictions to be flagged and enabling assessment of when a model can be trusted for a given prediction or design space.^35–37^ Previous studies have indicated that ensemble methods provide an effective and practical route to uncertainty quantification.^38,39^ By combining predictions from MLIPs with distinct initialization, architectures, and/or training data, ensembles provide a practical proxy for epistemic uncertainty. Most commonly, high error is expected for prediction on samples that are quite distinct from the training data points.

To correct systematic biases and estimate per-prediction uncertainty, we construct an ensemble of MLIPs to predict adsorption energies. We focus on OH adsorption as a key descriptor for oxygen reduction reaction (ORR)^40^ activity on heterogeneous catalysts. We observe a systematic deviation between ensemble predictions and DFT values taken from literature data sets using different computational protocols. Using a bias-correction method adapted from economic and weather forecasting,^41,42^ we improved the ensemble’s root mean squared error (RMSE) by 60% on a bimetallic evaluation data set using only a single additional DFT adsorption energy. This approach thus enables multifidelity integration of DFT data. We further quantify ensemble uncertainty to assess prediction reliability and apply the framework to screen a large trimetallic design space, efficiently identifying a promising ORR candidate with minimal additional quantum calculations.

## Methods

### DFT Methods

The only new density functional theory (DFT) calculations performed in this work were those done as part of the screening (Section “Screening Data Set”). They were carried out using the Vienna Ab Initio Simulation Package (VASP),^43,44^ employing the projector augmented wave (PAW) method^45^ with the Perdew–Burke–Ernzerhof (PBE) generalized gradient approximation (GGA).^46^ Long-range dispersion interactions were included using the Tkatchenko–Scheffler method.^47^ A plane-wave cutoff of 400 eV was applied throughout, consistent with prior alloy-surface adsorption studies using VASP PAW–PBE and shown to yield adsorption energies stable to within a few tens of meV under stricter settings.^48^ Electronic occupancies were determined using Methfessel–Paxton smearing^49^ with a width of 0.2 eV, and the self-consistent field convergence criterion was set to 10^–5^ eV. Structural relaxations were performed using a conjugate-gradient scheme,^44^ with the bottom two layers of the slab fixed at their bulk positions, and continued until the maximum residual force on any atom was below 0.03 eV/Å.

During the screening, the Brillouin zone was sampled using a Γ-centered Monkhorst–Pack grid^50^ of 4 × 4 × 1 *k*-points for the 3 × 3 × 4 surface cell. This choice was made to reduce computational cost in case a large number of calculations were needed. To validate that the reduced *k*-point sampling did not affect the screening conclusions, we recomputed adsorption energies for Pt and the final selected candidate using a denser 7 × 7 × 1 *k*-point grid. While the absolute adsorption energy of Pt shifted by 0.04 eV relative to the 4 × 4 × 1 calculations, the final candidate remained within ±0.1 eV of the target adsorption energy, defined as 0.1 eV weaker than Pt, and thus still met our screening criterion.

For gas-phase reference energies, we followed the Open Catalyst Project (OCP) convention, in which reference values are obtained as linear combinations of DFT energies of closed-shell molecules.^51,52^ Within this framework, the gas-phase energy of OH was defined as – 10.681 eV.

All other data sets used in this work (see “Evaluation Data Sets”) were taken directly from the literature and retain their original DFT computational protocols, which are described in their respective references.

### Ensembling

Our ensemble consists of five publicly available, pretrained MLIPs trained on OCP data: DimeNet++,^53^ SchNet,^54^ PaiNN,^55^ SCN,^56^ and GemNet.^57^ These models were selected because they (i) predict adsorption-energy targets and atomic forces directly from the adsorbed atomic structure within a common Structure-to-Energy-and-Forces (S2EF) framework, eliminating the need for explicit clean-surface or gas-phase reference calculations at inference time, (ii) span a diverse set of message-passing and equivariant neural-network architectures while sharing a consistent training distribution, and (iii) are interoperable within a single Fairchem-core (v1.10.0) software stack,^51^ enabling consistent ensemble evaluation across data sets. Although OCP-trained models are not universal foundation MLIPs, their training distribution is well aligned with surface–adsorbate chemistry, making them an appropriate foundation for studying few-shot adaptation under distribution shift.

Ensemble predictions were computed as the mean of the individual MLIP outputs, and the ensemble standard deviation was used as an estimate of predictive uncertainty. In the extreme few-shot regime considered here, obtaining reliably calibrated uncertainty estimates, whether via Bayesian or non-Bayesian approaches, is inherently challenging.^58,59^ Accordingly, this uncertainty estimate is interpreted as a pragmatic indicator of model disagreement rather than as a source of formal probabilistic guarantees.

More rigorous uncertainty quantification methods, such as Bayesian deep ensembles, typically require access to training data and the ability to retrain multiple independently initialized models,^60^ which introduces substantial additional complexity. In contrast, our approach uses pretrained models without modification, which lowers the barrier to adoption, enables immediate use without specialized training, and allows newly developed MLIPs to be incorporated as they become available.

### Bias Correction

Machine learning models trained on one DFT setup can exhibit systematic deviations when applied to data generated under a different computational protocol. To mitigate this, we adapt a bias-correction technique developed in economic forecasting.^41^ In its original context, this approach was used to evaluate and correct systematic prediction bias for a single regression model by subtracting the mean residual from its forecasts. In our study, we extend this idea to the MLIPs within our ensemble.

For a given structure, we obtain the predictions from each MLIP and compute the residuals relative to the DFT reference value. As additional reference data becomes available in the few-shot setting, we track each model’s systematic bias by averaging its residuals across the available reference structures. These per-model mean residuals are then used to shift the corresponding MLIP predictions before forming the ensemble. Although more expressive recalibration schemes, such as full linear correction of both slope and intercept, are possible, we find that such approaches become unstable in the extreme low-data regime considered here (see Supporting Information “Few-Shot Linearization”).

### Data Sets

#### Evaluation Data Sets

We adapted a data set of OH adsorption energies on bimetallic alloys (denoted OH-BMA) from Mamun et al.,^61^ comprising FCC-derived alloy structures built from combinations of 37 metals, with adsorption energies computed using the BEEF-vdW functional within Quantum ESPRESSO 5.1. Although the structures were already relaxed with DFT, we rerelaxed them using the UMA foundation model^62^ to better mimic a true screening process. The UMA model has demonstrated strong performance on surface systems. In accordance with the Mamun et al. study, bulk atoms below the first layer were held fixed to approximate bulk conditions.^63^

The rerelaxed structures were compared against their DFT references to identify cases where the DFT and MLIP converged to the same local minimum. A total of 406 structures in which the adsorbate migrated to a different binding site (e.g., bridge → hollow) were removed using PyMatGen routines.^64^ In these cases, the MLIP-relaxed and DFT-relaxed structures do not correspond to the same adsorption geometry and therefore cannot be meaningfully compared. The impact of retaining these anomalous relaxations on model performance is examined in the Supporting Information (Section “Anomalous UMA Relaxations”). In a screening context, structures where the MLIP relaxes away from the intended site would likely be discarded as unstable or would require additional constraints. By removing cases where the DFT relaxation and ML relaxation land in different basins, we ensure that our evaluation focuses on differences within shared minima rather than on discrepancies in binding configuration.

Note that the OH-BMA data set was generated using a different DFT setup than the MLIPs’ training data. This intentional discrepancy allows our evaluation to probe the method’s robustness to differences in computational setup. The resulting data set contained 829 labeled samples for testing the few-shot method.

In addition to the OH-BMA data set, we also evaluated our approach on a single-atom-alloy data set^48^ (denoted here as H-SAA), consisting of 85 hydrogen adsorption energies on FCC(111) coinage-metal surfaces with isolated dopant atoms drawn from 19 d-block metals. This data set was generated using the same DFT setup described in the “DFT Methods” section, but represents a distinct materials space from the OC20 training data used to train the MLIPs (SAAs versus intermetallic surfaces).

#### Screening Data Set

For our target screening space, we generated 3 × 3 × 4 trimetallic surfaces spanning a composition space of four host metals (Pt, Pd, Ag, Au) alloyed with 22 surface dopants (see Supporting Information, “Trimetallic Composition Space” section). To construct these systems, we doped only the surface layer and enumerated all unique dopant configurations, resulting in two classes of structures: dual-atom alloys (denoted (D_1_D_1_ *′*)­H), where the two dopants occupy adjacent sites forming a heterodimer,^65^ and dual single-atom alloys (denoted (D_1_+D_1_ *′*)­H), where the dopants are separated by at least one host atom.^66^ For example, (Pt_1_Cu_1_)Au corresponds to a dual-atom alloy, while (Pt_1_+Cu_1_)Au represents a dual single-atom alloy, both in a Au host. For each surface, we enumerated all unique OH binding sites and placed an OH adsorbate at those locations. The structures were relaxed using the same UMA model described above, and duplicates were removed using PyMatGen routines. The final screening space comprised 15,784 unique OH adsorption configurations.

### Few-Shot Campaign Strategy

Our objective in this work is to minimize the number of DFT calculations required to identify a promising candidate in a given materials design space. To achieve this, we propose a search strategy that iteratively applies DFT both to validate and refine the predictions of the MLIP ensemble. The procedure begins by generating zero-shot ensemble predictions, *ŷ*, and the corresponding ensemble standard deviations, σ, for all candidates in the screening data set. We then define a cost function that balances proximity to a target adsorption energy and predictive uncertainty:

$$\mathrm{Cost}(y_{\mathrm{target}},\hat{y},\sigma )=\alpha \frac{\hat{y}-y_{\mathrm{target}}}{{\hat{y}}_{\max}-{\hat{y}}_{\min}}+(1-\alpha )\frac{\sigma }{\sigma_{\max}-\sigma_{\min}}$$ 1

Here, *ŷ*_max_ and *ŷ*_min_ denote the maximum and minimum ensemble-predicted adsorption energies, respectively, evaluated over all candidates in the current screening data set. Similarly, σ_max_ and σ_min_ correspond to the maximum and minimum ensemble standard deviations across the same candidate set. These quantities are used to normalize both terms of the cost function, ensuring that the accuracy and uncertainty contributions are dimensionless and comparable in magnitude.

The parameter α controls the relative weighting between predicted proximity to the target value, *y*_target_, and predictive uncertainty. For the trimetallic data set campaign, we set α = 0.75, thereby biasing the search toward candidates with predicted adsorption energies close to the target while still penalizing high-uncertainty predictions.

In the zero-shot case, the process begins by selecting the structure with the lowest cost function value from the screening data set. Two DFT geometry relaxations, one for the adsorbed surface and one for the clean surface, are then carried out to obtain the adsorption energy with respect to the appropriate gas-phase reference (see Section “DFT Methods”). This adsorption energy is incorporated into the *n*-shot bias correction method to update the mean residual, improving the reliability of subsequent predictions. The corrected ensemble predictions are then used to rerank candidates according to the cost function, and the process is repeated iteratively until a promising candidate is discovered.

If a reference calculation has already been performed based on the chosen design principle, the workflow can use that reference value to begin directly with one-shot ranking rather than zero-shot ranking. For the screening study in this work, the reference system consisted of OH adsorbed at the bridge site on Pt(111). This reference adsorption energy was used both to define the target value and to initiate candidate ranking with the one-shot method.

## Results and Discussion

### Few-Shot Effect on MLIP and Ensemble Predictions

We first evaluated the few-shot bias-correction strategy, which updates ensemble predictions using only a handful of new DFT labels, to correct systematic deviations between the training data and a target data set. Briefly, the method shifts each MLIP prediction based on the new DFT labels (see “Methods” for more details). The evaluation was performed on the OH-BMA data set of 829 OH adsorption energies on bimetallic alloys^61^ (see “Evaluation Data Sets” section). In this case, both the original training data and the target data set are intermetallics, with some difference in the distribution of materials as well as a different computational setup.

As shown in Figure 1a, the constituent MLIPs (DimeNet++, SchNet, PAINN, SCN, and GemNet) exhibit moderate variation in zero-shot performance, with RMSE values ranging from 0.385 to 0.610 eV. Most models overestimate adsorption energies relative to DFT, indicating a shared systematic bias, which is inherited by the ensemble average (Figure 1b, blue circles). Applying the 5-shot bias correction (Figure 1b, orange triangles) shifts the ensemble predictions toward parity, significantly improving the RMSE to parity with DFT (RMSE_parity_) from 0.505 to 0.148 eV.

**Figure 1:**
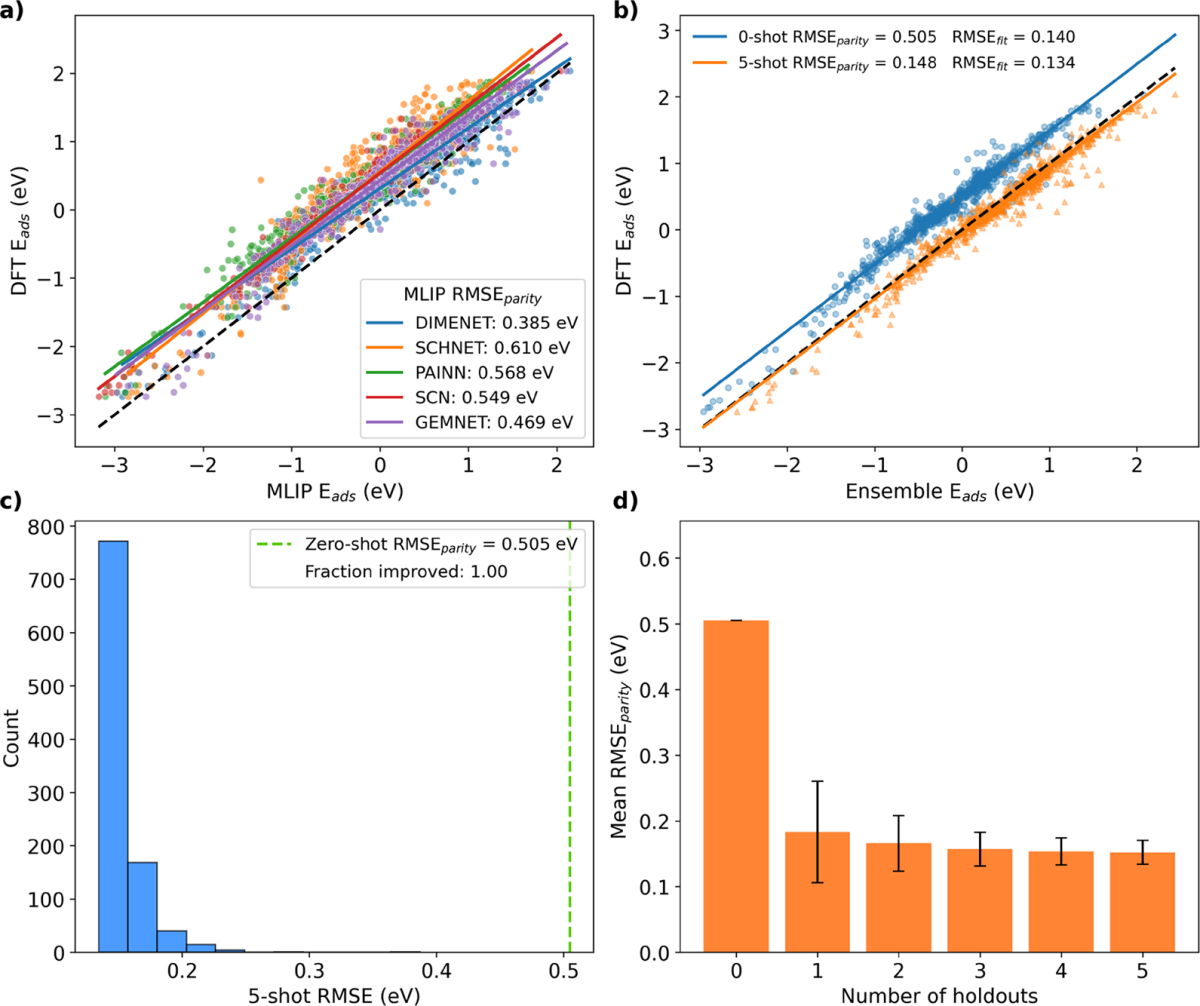
Few-shot learning on the OH-BMA data set, which primarily reflects a shift in computational setup from the original training data. (a) DFT vs MLIP adsorption energies. Each MLIP is strongly correlated with DFT. (b) DFT vs MLIP ensemble-mean adsorption energies. A 5-shot bias correction substantially improves ensemble accuracy. (c) Histogram of MLIP ensemble RMSEs for different random sets of 5 data points in the 5-shot method. All yield lower RMSEs than the zero-shot baseline. (d) Mean RMSE, with standard deviations, for *n*-shot with varying *n*. RMSE decreases with more samples.

Consistent with the visual appearance of Figure 1b, the RMSE relative to the best-fit linear trend, which reflects residual scatter after trend alignment, changes only weakly from 0.140 to 0.134 eV. This indicates that the primary effect of the correction is the removal of a systematic energy bias rather than a large reduction in point-wise scatter, and this behavior is observed in Figure 2b as well. Hereafter, RMSE refers to the error computed relative to the DFT parity line.

**Figure 2:**
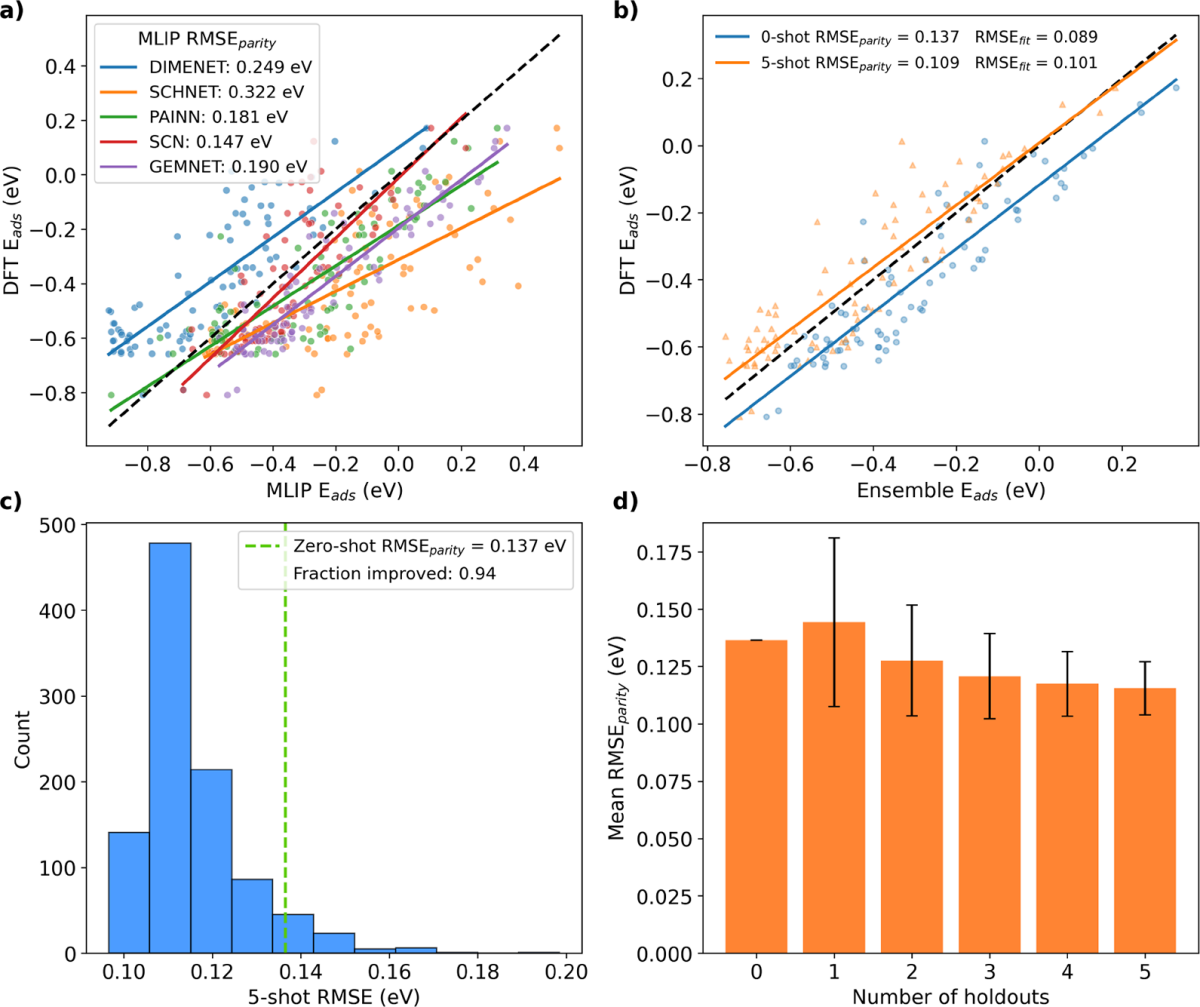
Few-shot learning on the H-SAA data set,^48^ representing a shift of computational setup and materials space from the original training data. (a) DFT vs MLIP adsorption energies. The MLIPs show low-to-moderate correlation with one another and with DFT. (b) DFT vs MLIP ensemble-mean adsorption energies. Even the zero-shot ensemble is more accurate than any individual MLIP. (c) Histogram of MLIP ensemble RMSEs for different random sets of 5 data points in the 5-shot method. Most yield lower RMSEs than the zero-shot baseline. (d) Mean RMSE, with standard deviations, for *n*-shot with varying *n*. Mean RMSE decreases steadily with more samples after early instability.

We attribute this bias primarily to differences in the DFT parameters used to generate *y*_train_ and *y*_test_. This interpretation is supported by control experiments in which the training and test data were generated using the same DFT setup; in that case, no systematic bias or improvement from bias correction was observed (see Supporting Information, “OC20 Baseline” section).

Depending on which samples are selected for the *n*-shot method, the resulting RMSE varies, as shown in Figure 1c, which reports results from 1000 independent, random 5-shot combinations. All combinations yield a lower RMSE than the zero-shot baseline (0.505 eV). To assess how performance scales with sample count, we computed the mean RMSE across bias-correction runs using zero to five training samples (Figure 1d). The largest improvement occurs after the first added sample, with the RMSE dropping by roughly 0.3 eV on average, while subsequent samples lead to diminishing yet consistent reductions in both mean RMSE and variance. Beyond five added samples, performance gains begin to saturate (see Supporting Information, “Extended Few-Shot Regime” section). We further demonstrate the generality of the few-shot bias-correction framework by applying the same analysis to additional adsorbates (NH and O) in the Supporting Information (“Additional Adsorbates” section).

We next evaluate how rapidly the ensemble can learn an appropriate bias correction for a target data set drawn from a chemical space that differs somewhat from that used to train the MLIPs. To this end, we apply the method to a data set for hydrogen adsorption on single-atom alloys (H-SAA),^48^ which represents a distinct class of catalytic materials that differs from both the OC20 training data and the OH-BMA data set. In contrast to the OH-BMA data set shown in Figure 1a, for which the MLIP predictions consistently exhibit overbinding relative to DFT, the MLIP predictions for the H-SAA data set (Figure 2a) span a broader range of behaviors, including overbinding, underbinding, and mixed residuals across models. As a consequence of this distribution of model behaviors, the ensemble-averaged prediction remains reasonably correlated with the DFT reference energies but exhibits a systematic underbinding bias (Figure 2b). In this case, the zero-shot ensemble is more accurate than any individual MLIP. As shown in Figure 2c, across 1000 random sample combinations used in the 5-shot procedure, there is about a 95% probability of improving upon the zero-shot baseline. For the H-SAA data set, the one-shot method initially increases the RMSE on average before steadily decreasing as more samples are added (Figure 2d). To better understand the behavior of the MLIPs forming the ensemble, we performed a per-model analysis on the H-SAA data set (see Supporting Information, “MLIP Subsets” section).

### Uncertainty Quantification

To assess the reliability of a given ensemble prediction, we use the standard deviation across models as a per-sample measure of uncertainty. Uncertainty quantification typically employs frameworks explicitly designed to introduce model variation. In contrast, we construct our ensemble from models developed independently in prior studies, which motivates an evaluation of how effectively this type of ensembling captures prediction reliability. We observe that predictions with larger standard deviations tend to be less accurate (see Figure 3a), indicating that the ensemble’s spread serves as a reasonable proxy for uncertainty. This analysis uses the same 5-shot ensemble shown in Figure 1b,c. We further evaluate our ensemble’s uncertainty quantification using metrics and visualization frameworks proposed previously.^35^ This qualitative comparison gives context for the ensemble’s uncertainty quantification, although absolute values cannot be quantitatively compared due to variations in the data set between our work and the previous work.

**Figure 3:**
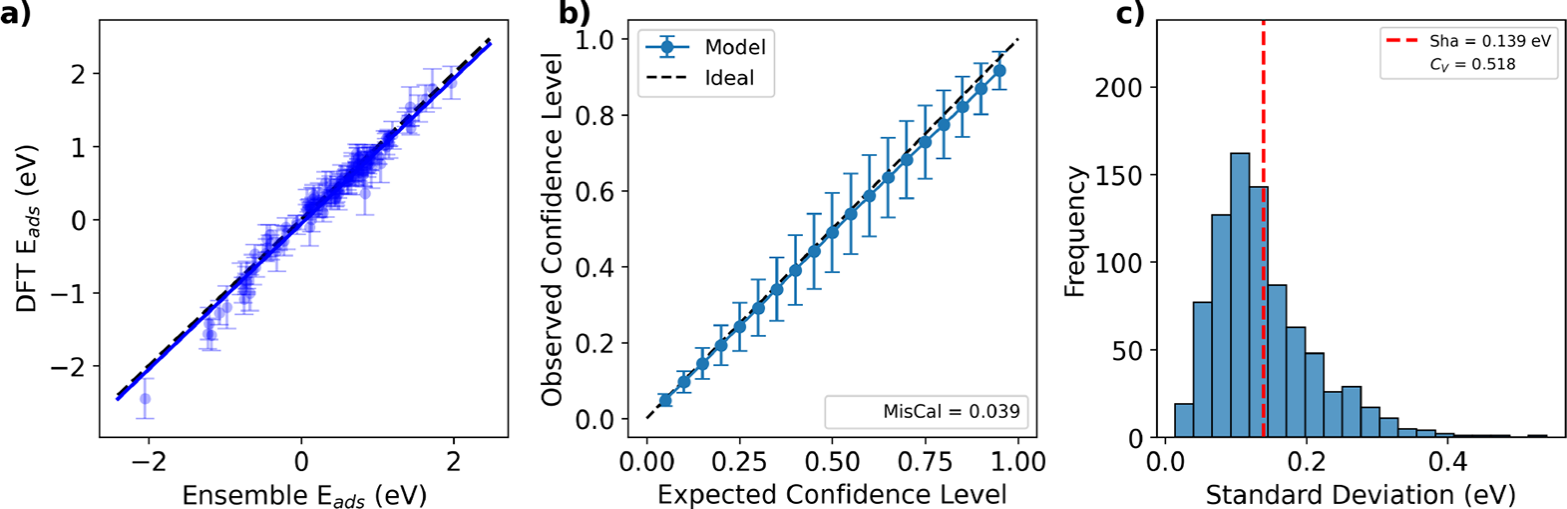
5-shot uncertainty quantification on the OH-BMA data set. (a) Predictive parity plot with uncertainty predictions as error bars. (b) Observed vs expected confidence levels. Predicted confidence intervals correspond closely to actual coverage. (c) Histogram of uncertainty values. The ensemble uncertainties are relatively small and moderately diverse across the data set.

The calibration curve and its associated miscalibration area quantify the alignment between predicted confidence and observed accuracy (Figure 3b). The error bars represent variation across 1000 random combinations of five training samples in the 5-shot method. The ensemble demonstrates strong calibration, closely following the ideal diagonal with a miscalibration area of only 0.039, compared to 0.12 for the neural network ensemble reported in the literature.^35^ This indicates that the ensemble’s uncertainties provide a reliable measure of confidence in adsorption-energy predictions.

Calibration assesses whether predicted confidence intervals are statistically consistent with observed errors, but it does not describe their overall magnitude. We first examine sharpness, which reflects the overall width of the predicted confidence intervals. Lower sharpness values are desirable, as narrower confidence intervals indicate more precise predictions. Figure 3c shows the distribution of ensemble standard deviations on the OH-BMA data set, yielding a sharpness of 0.139 eV, which is smaller than the literature value (0.16 eV).^35^ Because sharpness can be evaluated without reference data, it is also particularly well suited as a practical diagnostic for determining when additional bias-correction samples are warranted; further analysis of sharpness as a stopping criterion for the few-shot procedure is provided in the Supporting Information (Section “Extended Few-Shot Regime”).

To complement sharpness, we next consider the coefficient of variation (*C*_V_), which quantifies how much the predicted uncertainties vary across samples. Higher *C*_V_ values indicate greater heterogeneity in the uncertainty estimates, reflecting more differentiated model confidence across the data set. For the OH-BMA data set, the ensemble yields a *C*_V_ value of 0.518, which is smaller than the literature reference value of 1.06.^35^ One possible explanation for this reduced variability is the shared training data among the FairChem MLIPs used in this study, leading to correlated model behavior when applied to new intermetallic surfaces. Overall, while the ensemble produces reasonably sharp and informative uncertainty estimates, the lower variability in uncertainty suggests an opportunity to further increase model diversity.

We also assessed the utility of the uncertainty quantification using the H-SAA data set. As shown in Figure 4a, samples with wider prediction intervals tend to exhibit larger deviations from the parity line, indicating that the reported uncertainties meaningfully reflect prediction accuracy. This is corroborated by panel b, where the calibration curve follows near-ideal behavior with a low miscalibration area (0.088). Finally, the distribution of ensemble standard deviations in panel c shows a sharpness of 0.110 eV, smaller than the literature value (0.16 eV), and a moderate coefficient of variation (*C*_V_ = 0.471), indicating that the ensemble shows a moderate degree of variability in its uncertainty estimates. Because the H-SAA data set is similar to the trimetallic screening space we are targeting, this evaluation provides a strong indication that the ensemble’s uncertainty estimates will remain reliable under the conditions most relevant to the screening campaign.

**Figure 4:**
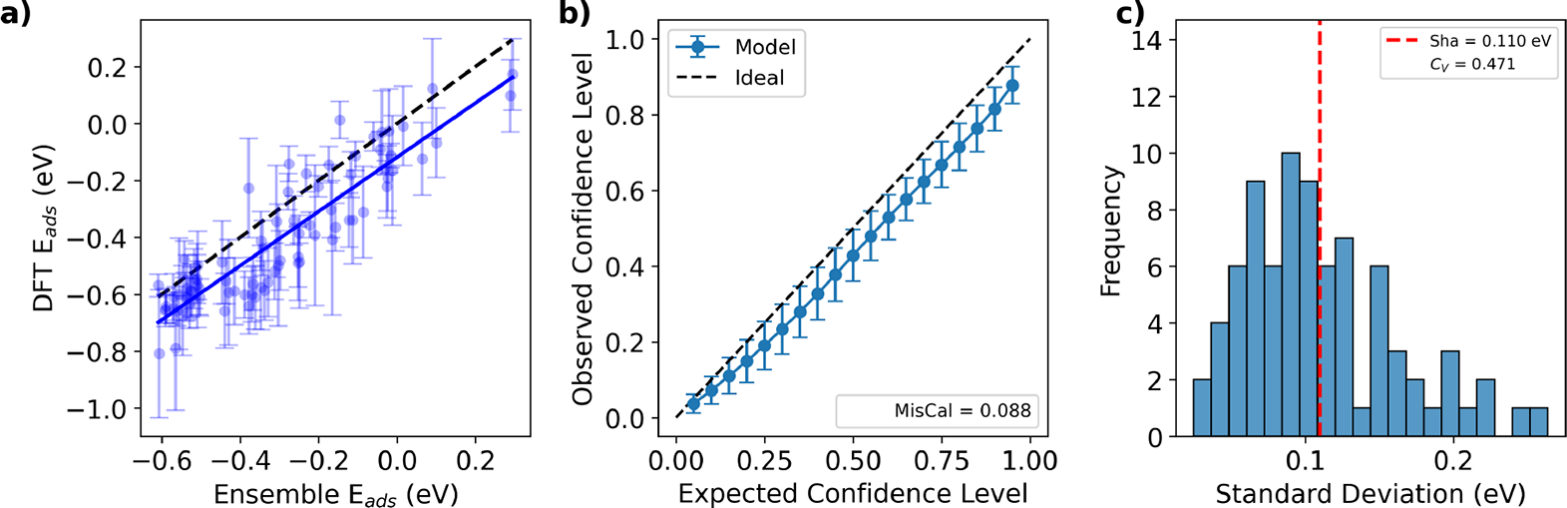
5-shot uncertainty quantification on the H-SAA data set. (a) Predictive parity plot with uncertainty predictions as error bars. (b) Observed vs expected confidence levels. Predicted confidence intervals correspond closely to actual coverage. (c) Histogram of uncertainty values. The ensemble uncertainties are relatively small and moderately diverse across the data set.

One significant advantage of uncertainty quantification is that it can help identify when model predictions are unreliable before committing to a screening campaign. To this end, we evaluate the zero-shot uncertainty quantification on three data sets: the OH-BMA and H-SAA data sets (see “Evaluation Data Sets” section), and a graphene single-atom catalyst (Graphene-SAC) data set^67^ obtained by web scraping from the ioChem-BD platform.^68^ The Graphene-SAC data set represents a markedly different chemical domain, serving as an out-of-distribution test case since it involves metallic dopants binding to organic substrates rather than organic adsorbates on metals. For consistency across data sets, we excluded the DimeNet++ model from the ensembles, as it produced unphysically large predictions for the Graphene-SAC data set. For completeness, we also report the uncertainty quantification of the trimetallic screening data set; in this case, we retained DimeNet++ in the ensemble, since it was used in the actual screening campaign.

We hypothesized that the ensemble’s sharpness (i.e., average per-sample uncertainty) may give an indication of model reliability; see Table 1 for the sharpness and model accuracies. The Graphene-SAC data set exhibits substantially higher sharpness than the other two data sets, reflecting much greater disagreement among ensemble members. This elevated uncertainty correctly signals that the model is operating far outside its training distribution, leading to poor predictive accuracy with a very high RMSE of 2 eV and a poor *R* ^2^ value of 0.43. Even for the alloy data sets, the sharpness correctly suggests that the OH-BMA data set would give a higher *R* ^2^ value (0.97) as compared to the H-SAA data set (0.80). (We attribute the lower RMSE for the H-SAA data set to the lower variation in energies due to H’s weaker binding.)

**Table 1:** Zero-Shot Accuracy (via RMSE), Correlation with DFT Energies (via *R* ^2^), and Estimated Average Uncertainty (via Sharpness) across Data Sets

data set	0-shot RMSE (eV)	*R* ^2^	sharpness (eV)
OH-BMA	0.532	0.97	0.131
H-SAA	0.176	0.82	0.113
graphene-SAC	2.061	0.43	0.832
trimetallic	N/A	N/A	0.106

Importantly, while RMSE and *R* ^2^ can only be computed once all test labels are known, sharpness requires no reference data. It therefore provides a true zero-shot diagnostic of model trustworthiness: when ensemble predictions are too broad to resolve chemically meaningful trends, the models should be treated as unreliable. Our analysis of the trimetallic data set in Table 1 serves as such a diagnostic, demonstrating that the ensemble operates within a reliable regime and that our screening campaign is likely to be effective. At the level of individual predictions, ensemble disagreement can also act as a diagnostic tool, flagging specific subsets of structures that warrant closer inspection; an example involving O adsorption on lanthanum-containing alloys is discussed in the Supporting Information (“Additional Adsorbates” section).

### N-Shot Search Campaign

Our application of interest was the ORR, for which the OH binding energy is widely used as a descriptor of catalytic performance.^40^ Pt is the industrial benchmark catalyst, and prior theoretical studies have indicated that catalysts with slightly weaker OH binding than Pt should exhibit enhanced ORR activity.^69,70^ We use this as our design principle for this study, although our method could be readily applied to more complex design principles using multiple adsorbates.^71^

To demonstrate the practical application of the ensemble’s bias correction and uncertainty quantification, we carried out an iterative search campaign on our unlabeled trimetallic data set of dual-atom alloys and dual single-atom alloys (see “Screening Data Set” section). As a reference, we used DFT to compute the OH binding energy on a four-layer Pt(111) slab, obtaining a value of 0.68 eV. Since the theoretically optimal ORR catalyst binds OH roughly 0.1 eV more weakly than pure Pt,^70^ we set our target binding energy to 0.78 eV. The campaign was terminated once a candidate surface exhibited a DFT-calculated OH adsorption energy within ± 0.1 eV of this target. Because the DFT calculation for OH adsorption on Pt was already available, it served as the initial sample in our *n*-shot method, and subsequent DFT calculations were used to iteratively update the ensemble predictions based on the cost function (eq 1).

Our search methodology initially selected the dual-atom alloy (Cu_1_Ir_1_)Au as the top candidate. However, upon DFT relaxation, the adsorbate migrated to a different binding site, rendering the corresponding ML ensemble adsorption energy prediction inapplicable. Consequently, the next highest-ranked system, (Au_1_Mn_1_)Ag (Figure 5), was used as the next sample in the bias-correction process. This candidate did not yield a DFT-calculated adsorption energy within the target range. After incorporating this result and performing a second update, the ensemble identified the dual-atom alloy (Ag_1_Rh_1_)Au as the next candidate. The DFT-calculated adsorption energy for this system was 0.71 eV, just 0.07 eV from the target, marking the end of the campaign.

**Figure 5:**
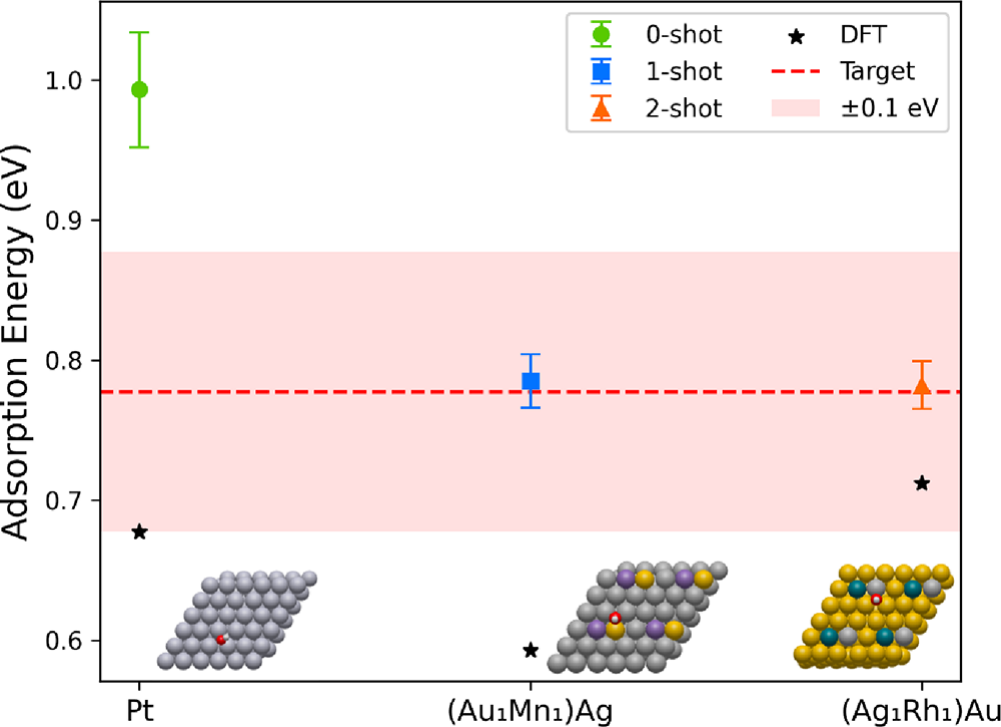
Iterative bias correction during the trimetallic search campaign. Successive updates guide the ensemble toward the target adsorption energy, identifying (Ag_1_Rh_1_)Au as a promising ORR candidate after two shots.

To better interpret the ensemble’s adaptive behavior, we applied the zero-shot, 1-shot, and 2-shot bias-correction updates post hoc to all three systems (see Supporting Information, section Posthoc Campaign Analysis). The 1-shot correction on Pt exactly reproduces the DFT value, since only a single residual is available for calibration, while the 2-shot prediction remains more accurate than the zero-shot prediction. For (Au_1_Mn_1_)­Ag, the accuracy steadily increases such that the 1-shot prediction is (incorrectly) within the target range, while the 2-shot prediction is closer to the correct adsorption energy. Similarly, the accuracy for (Ag_1_Rh_1_)Au steadily improves, yielding a prediction that meets the target criterion defined for this campaign.

In addition, we assessed the sensitivity of the candidate ranking to the weighting parameter α in the cost function (see eq 1). The initially selected top candidate remained first-ranked over a broad range of α values (0.35–0.95). Following its exclusion due to DFT relaxation failure, the next highest-ranked system, (Au_1_Mn_1_)­Ag, was consistently ranked second across α values spanning 0.40–0.95. After incorporating this result, the subsequent candidate, (Ag_1_Rh_1_)­Au, retained its ranking over a similarly wide range (α = 0.65–0.85). These results indicate that the campaign trajectory was robust to substantial variation in α and was not driven by fine-tuned weighting of the cost-function parameters.

Thus, using existing ML models, we were able to efficiently screen a new design space of trimetallic surface alloys, distinct from the intermetallic surfaces in the original training set and with a different computational setup. This allowed identification of a promising ORR candidate, predicted to be more active than Pt based on a simple and widely used design principle, with only four DFT-calculated adsorption energies.

## Conclusions

MLIPs have become powerful tools for accelerating catalyst discovery, but they are generally difficult to use as black-box models across diverse applications due to systematic biases and poorly quantified uncertainty. Differences in DFT methodologies or material spaces further complicate their use in practical screening. Given these issues, efficiently leveraging existing MLIPs requires methods that can adapt models with minimal additional data while quantifying prediction confidence to guide trust and data collection. Ideally, such methods would require little specialized expertise or additional computational effort by users.

To address these challenges, we developed a framework that applies a few-shot bias correction to an ensemble of existing pretrained models using only a very small number of additional DFT calculations performed with the target computational setup in the target design space. Using just a single additional DFT adsorption energy, the few-shot ensemble reduced the RMSE from roughly 0.5 eV to roughly 0.2 eV on average for a database of OH adsorption energies. On a database of H adsorption energies on single-atom alloys, even the zero-shot ensemble is more accurate than any single MLIP, likely due to the change in material space between the training and target data sets. The resulting uncertainty estimates are well-calibrated, while the coefficient of variation (*C*_V_ = 0.52) suggests possible improvements through heterogeneous training or ensemble construction in future work. The methodology also identifies cases where performance is likely to be poor without requiring any DFT calculations, helping prevent misuse of the models in inappropriate contexts. Finally, in a search campaign, it located a promising ORR candidate within just two adaptive iterations (four total DFT-calculated adsorption energies), illustrating its ability to accelerate catalyst screening with minimal computational expense.

This work demonstrates that few-shot bias correction can iteratively enhance the accuracy of ML predictions without modifying the underlying model architecture, enabling highly efficient screening. The proposed framework is intentionally modular and operates entirely on pretrained model outputs, allowing it to remain agnostic to the chemical complexity of the target system. As ML interatomic potentials continue to advance toward increasingly complex catalytic environments, the framework can readily incorporate these developments without retraining or architectural modification. In this way, existing models, including those trained on unrelated data sets or based on approximate physical formalisms (e.g., density functional tight-binding), serve as rapid, low-cost estimators of the target property space that can be efficiently refined with only a small number of additional reference calculations, even when the target search space differs from the original training domain.

## Supplementary Material



## Data Availability

Few-shot visualization code: https://github.com/SiliconScientist/transfer-shot.git. Trimetallic generation code: https://github.com/SiliconScientist/intergen.git.

## Notes

Analysis of anomalous UMA relaxations; definition of the trimetallic composition space; comparison of intercept-only and linear bias-correction schemes; OC20 baseline control experiments; few-shot results for additional adsorbates (NH and O); extended few-shot regime up to ten shots; MLIP subset analyses; and post hoc analysis of the trimetallic screening campaign (PDF)
