# Supplementary material for: Few-Shot Ensemble Learning for Catalysis and Application to Trimetallics for Oxygen Reduction
Source: ACS Catal. 2026 Mar 2;16(6):5592–602. doi: 10.1021/acscatal.5c08168 (PMC13010251; doi:10.1021/acscatal.5c08168)
Supplement: Supplementary file 1 [file cs5c08168_si_001.pdf]

Supporting Information for: Few-Shot Ensemble Learning for Catalysis and Application to Trimetallics for Oxygen Reduction

Avery F. Hill[1], Andrea Ruiz-Escudero[2,3], and Matthew M. Montemore[1,*]

[1]Department of Chemical and Biomolecular Engineering, Tulane University,, 6823 St. Charles Ave., New Orleans, LA 70118, USA
[2]Department of Computer Science and Information Technologies, Faculty of Computer Science, University of A Coruña, Campus de Elviña, A Coruña 15071, Galicia, Spain
[3]Department of Organic and Inorganic Chemistry, University of the Basque Country UPV/EHU, Barrio Sarriena s/n, Leioa 48940, Bizkaia, Spain

*Correspondence: mmontemore@tulane.edu

Anomalous UMA relaxations

In a subset of cases, the UMA relaxation led either to migration of the adsorbate away from the intended adsorption site or to unphysical structural distortions of the surface, such as significant displacement of surface atoms from the slab. In both situations, the resulting geometries are not comparable to the corresponding DFT-relaxed reference structures. While such configurations would likely be excluded in a practical screening workflow, we analyze them here to assess their effect on MLIP prediction quality and on the few-shot bias-correction procedure.

Considering only the anomalous cases substantially increases scatter relative to the main OH-BMA dataset (Figure S1), with RMSE values nearly double those reported in the main text. This degradation persists across individual MLIP predictions and the ensemble-averaged estimates, indicating that the few-shot correction is unable to compensate for the structural mismatch introduced by divergent relaxations.

These results support the exclusion of such anomalous relaxations from performance evaluation, as their inclusion would conflate errors due to model inaccuracies with discrepancies arising from fundamentally different adsorption geometries.

Trimetallic composition space

We selected Pt, Pd, Ag, and Au as host metals because they span a wide range of OH binding strengths and are electrochemically stable. Pt serves as the benchmark ORR catalyst, while Pd binds OH more weakly and is often alloyed with Pt to reduce cost and tune activity.[2] Ag and Au bind OH very weakly and are frequently used as inert hosts in single-atom alloy studies to isolate dopant effects.[3] All four hosts share the face-centered cubic (FCC) structure, allowing consistent comparisons on the (111) surface commonly used in ORR descriptor studies.[4]

We then selected 22 dopants to span a broad range of electronic and chemical diversity, including noble metals (Ag, Au, Pt, Pd, Rh, Ir), late transition metals (Cu, Ni, Co, Fe, Ru, Os, Mn, Re), and less-studied elements (Cr, Mo, W, V, Ta, Ti, Hf, Sc). This coverage captures known alloying strategies while enabling exploration of less conventional chemistries.[5, 6]

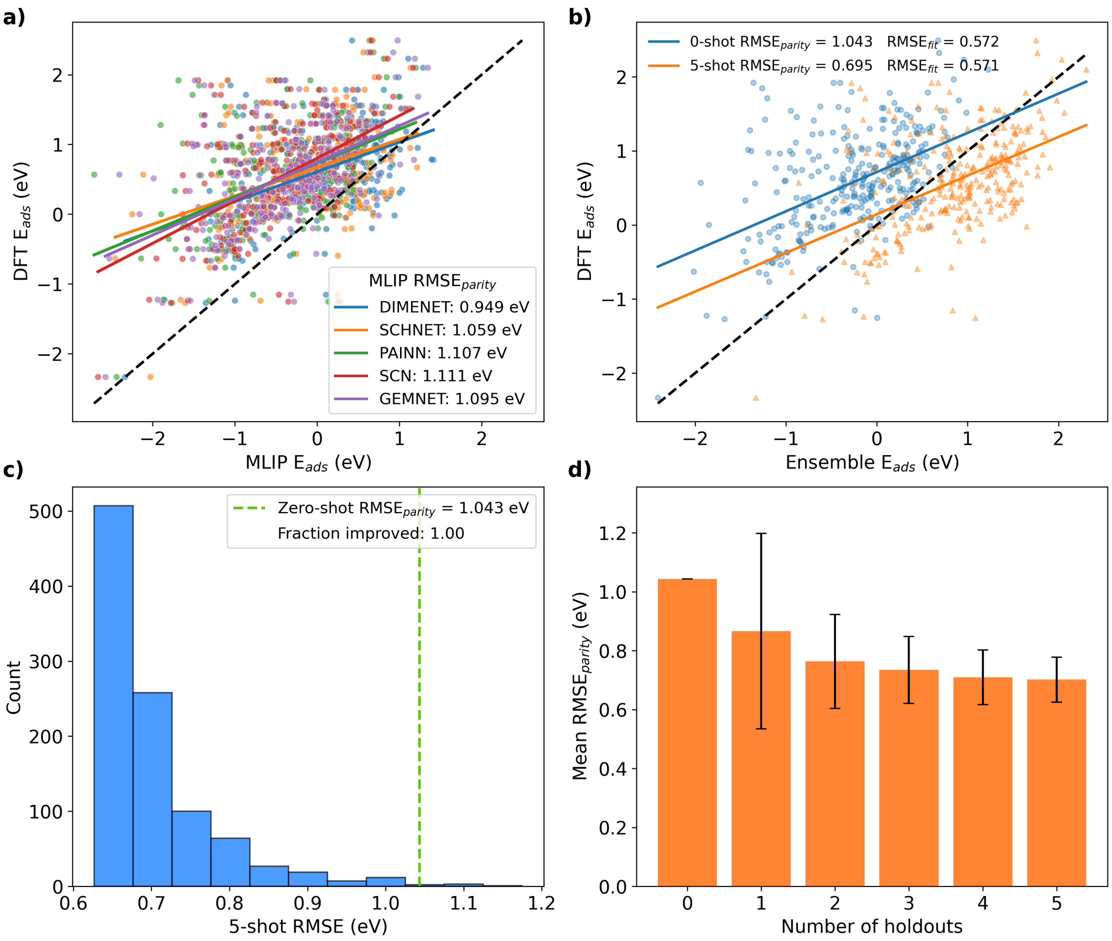

Figure S1: Few-shot performance on only the anomalous UMA relaxations from the OH-BMA dataset,[1] with a) MLIP parity plot, b) ensemble parity plot, c) histogram of 5-shot RMSEs, and d) RMSE vs. number of holdouts. Divergent relaxations lead to substantial scatter in MLIP and ensemble predictions, with RMSE values nearly double those observed for the corresponding OH-BMA analysis in the main text.

Few-shot linearization

The bias-correction method used in the main text is essentially an intercept shift, but it can be extended to modify both the intercept and the slope. We performed this linear scaling using the OH-BMA dataset described in the main text, **Evaluation Datasets** section, resulting in Figure S2.

The key differences between the intercept-shift and full linearization methods are that (1) linearization cannot be applied when only one data point is available, and (2) the initial 2-shot linearization produces highly variable RMSE values, often worse than the zero-shot baseline. As additional samples are incorporated, the RMSE values begin to resemble those from the few-shot intercept-shift method.

With a larger number of samples, the linearization approach might eventually outperform the intercept-shift method. Furthermore, this method could be highly useful in other cases

where a linear scaling between model predictions and DFT occurs. However, to address the few-shot regime, we focused on the intercept-shift approach for its data efficiency, robustness, and simplicity.

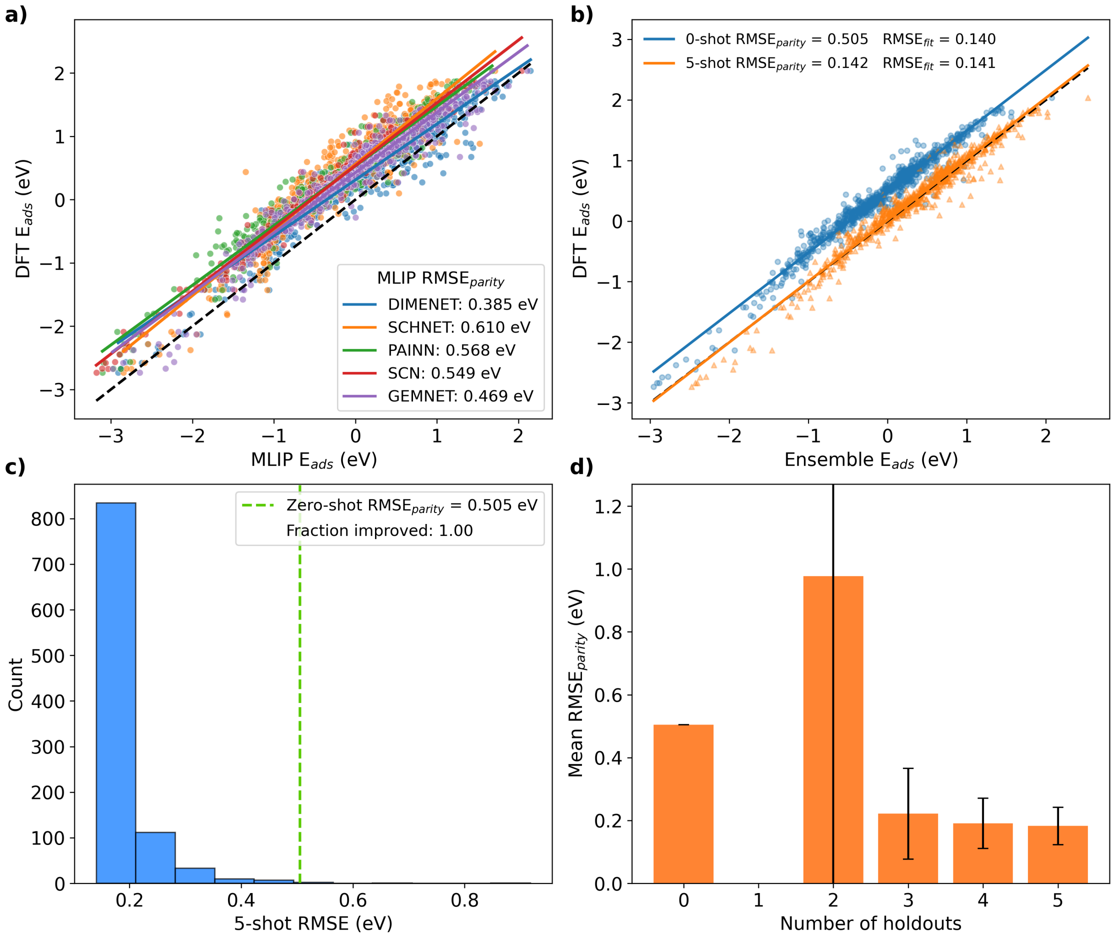

Figure S2: Few-shot ensemble performance on the OH-BMA dataset[1] using the linearization method. **a)** DFT adsorption energy vs. MLIP adsorption energy. **b)** DFT adsorption energy vs. MLIP ensemble adsorption energy. **c)** Histogram of MLIP ensemble RMSEs for different random combinations of 5 data points in the 5-shot method. **d)** Mean RMSE, with standard deviations, for n-shot with varying n. Mean RMSE shows early instability that smooths out and converges toward the intercept-shift results.

Additionally, we tested an outlier removal strategy, where individual MLIP predictions outside 1 standard deviation of the ensemble of predictions were removed. This slightly decreased accuracy for the OH-BMA dataset and slightly increased accuracy for the H-SAA dataset. Thus, while this could be useful in some use cases, particularly when a few of the models have low accuracy, we did not employ this strategy in this work.

OC20 baseline

We observed a bias between the OC20-trained MLIPs and the DFT reference adsorption energies in the OH-BMA dataset (see main text, **Evaluation Datasets** section). To identify the origin of this bias, we conducted a control experiment in which the training and reference data shared the same computational setup. Specifically, we filtered the OC20 out-of-domain (OOD) validation dataset for the OH adsorbate, yielding 450 surfaces. The OC20 dataset was generated using the RPBE exchange–correlation functional within VASP, providing a consistent reference for the MLIPs' training and evaluation. We then obtained zero-shot MLIP predictions for these systems and compared them with the OC20 DFT reference labels.

As shown in panels **a** and **b** of Figures S3, no systematic bias is present, and bias correction introduces negligible change in the RMSE. Furthermore, panel **c** indicates that there are few sample combinations that offer any advantage over the zero-shot baseline. Panel **d** suggests that the method is ultimately converging back to the original zero-shot RMSE. This control experiment demonstrates that the benefit of our few-shot bias correction method arises only when a systematic computational bias exists between the training and application datasets, or there is a change in the type of materials as for the H-SAA dataset. In the absence of such a bias, as in this OC20-to-OC20 comparison, the few-shot bias correction offers a limited accuracy advantage over the zero-shot baseline, although is likely still useful for quantifying uncertainty.

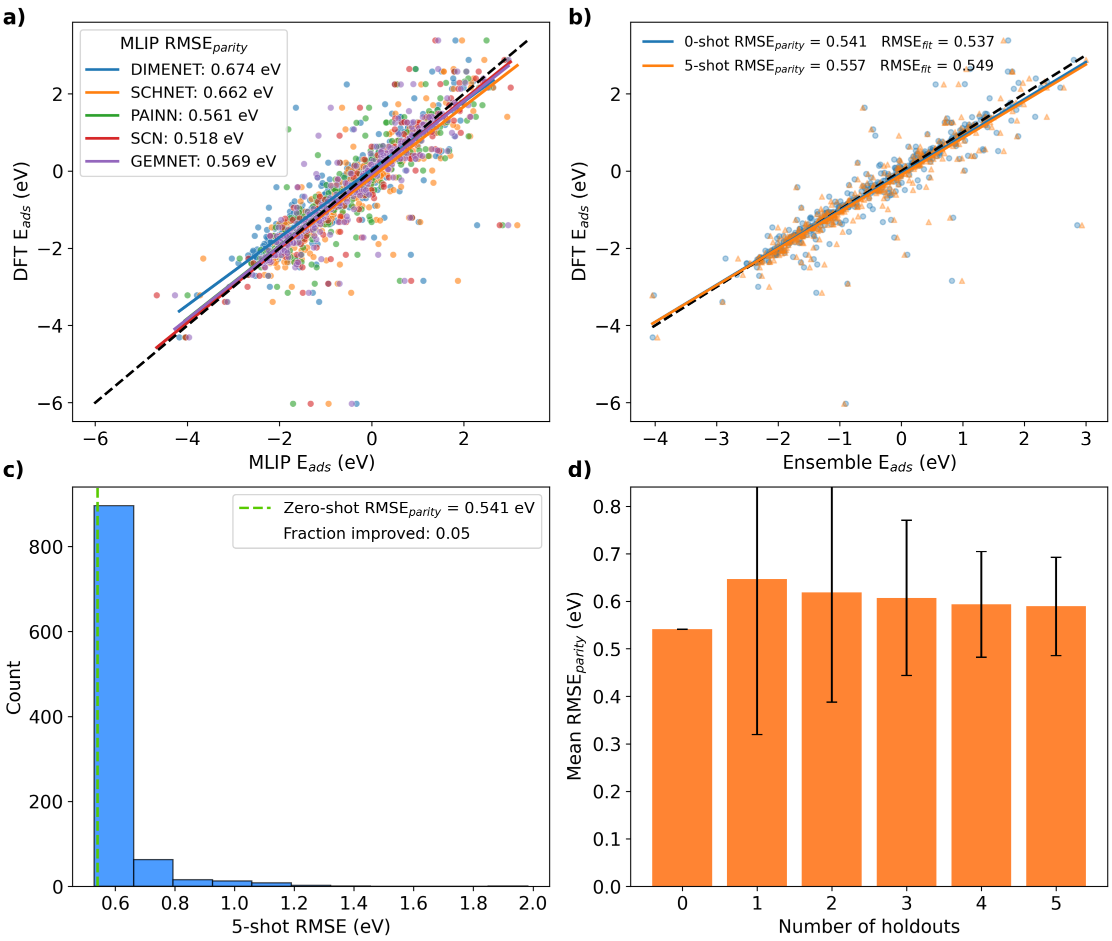

Figure S3: Few-shot ensemble performance on the OC20 dataset. **a)** DFT adsorption energy vs. MLIP adsorption energy. Individual MLIPs show consistent linear correlations with DFT values. **b)** DFT adsorption energy vs. MLIP ensemble adsorption energy. Few-shot correction has little effect on the RMSE relative to the zero-shot baseline. **c)** Histogram of MLIP ensemble RMSEs for different random sets of 5 data points in the 5-shot method. Most random holdouts fail to improve upon the baseline. **d)** Mean RMSE, with standard deviations, for n-shot with varying n. Increasing sample count offers no systematic improvement.

Additional adsorbates

To assess the generality of the few-shot bias-correction framework beyond OH adsorption, we applied the same analysis to additional adsorbates in the BMA dataset. NH (Figure S4) behaves similarly to OH, where few-shot updates lead to systematically improved agreement with DFT reference energies, indicating that the method transfers cleanly to this adsorbate without introducing qualitative changes in model behavior.

In contrast, analysis of O adsorption revealed a small number of extreme outliers accompanied by unusually large ensemble uncertainty. Indeed, for the 5-shot case, several predictions exhibit ensemble standard deviations exceeding 1 eV (Figure S5). Inspection of these

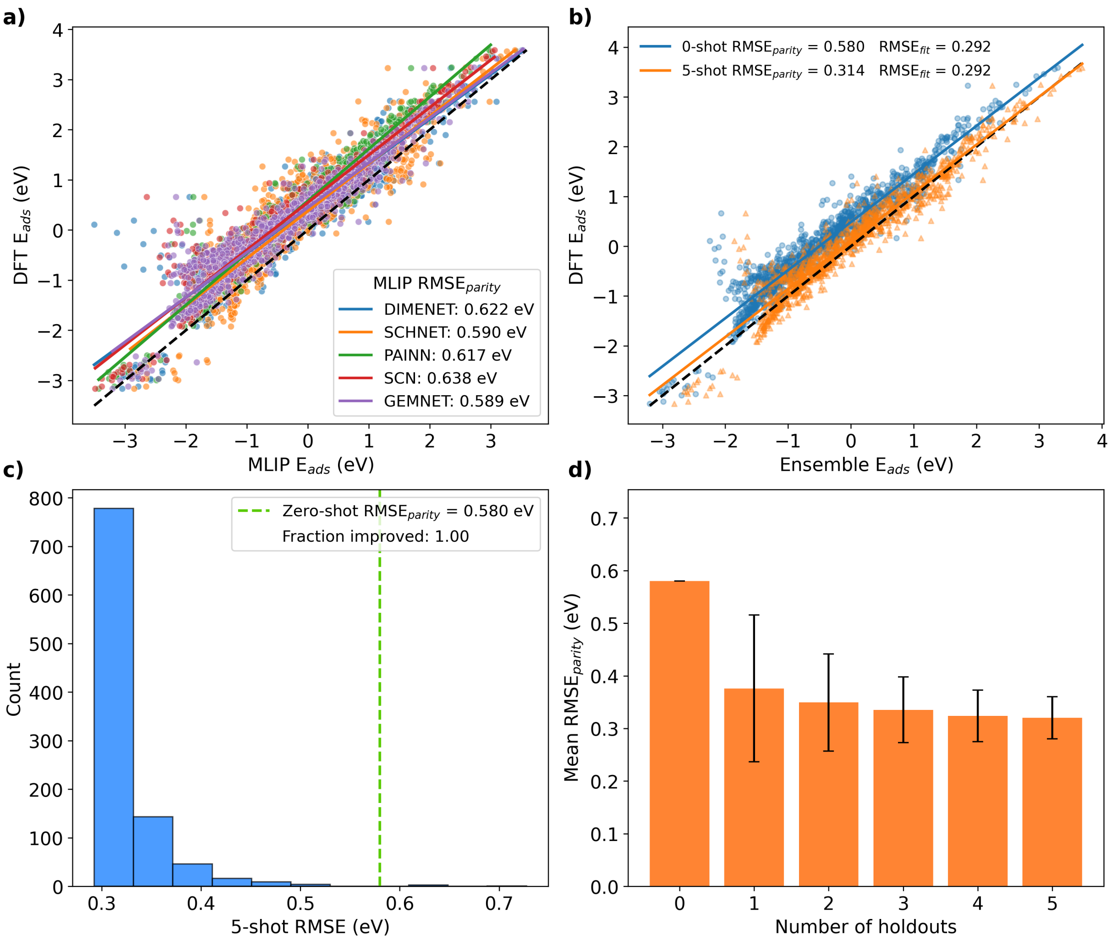

Figure S4: Few-shot ensemble performance on the NH-BMA dataset.[1] The bias-correction behavior mirrors that observed for OH-BMA in the main text

high-uncertainty structures revealed that all such cases contain lanthanum in the surface. This behavior is consistent with limited representation of lanthanum-containing systems in the training data and highlights a regime in which the constituent MLIPs strongly disagree. This analysis further illustrates how ensemble disagreement can serve as a diagnostic tool for identifying systems where the MLIPs are unreliable.

To isolate the effect of these samples, all lanthanum-containing structures were removed from the O-BMA dataset and the analysis was repeated (Figure S6). After lanthanum removal, the extreme outliers are eliminated, and the bias-correction behavior closely mirrors that observed for OH-BMA and NH-BMA, with improved accuracy, reduced ensemble RMSE, and monotonic improvement with increasing shot count.

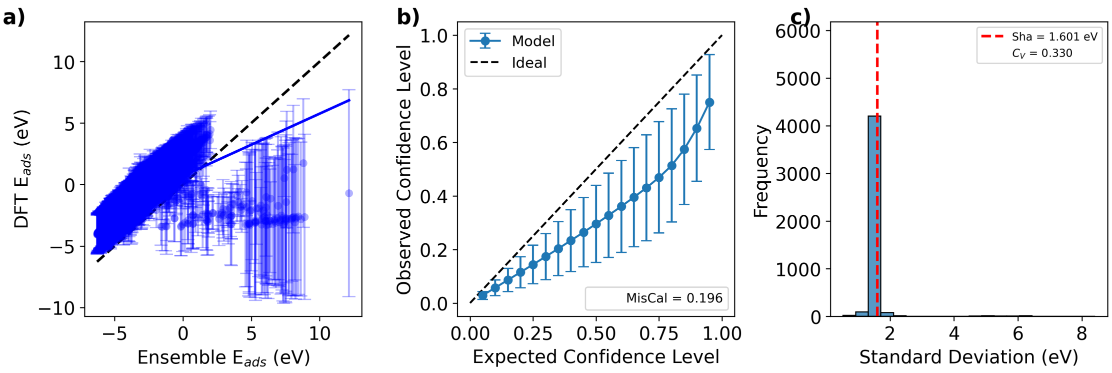

Figure S5: 5-shot uncertainty quantification on the O-BMA dataset.[1] The high uncertainty predictions in panel **a)** correspond to structures containing lanthanum.

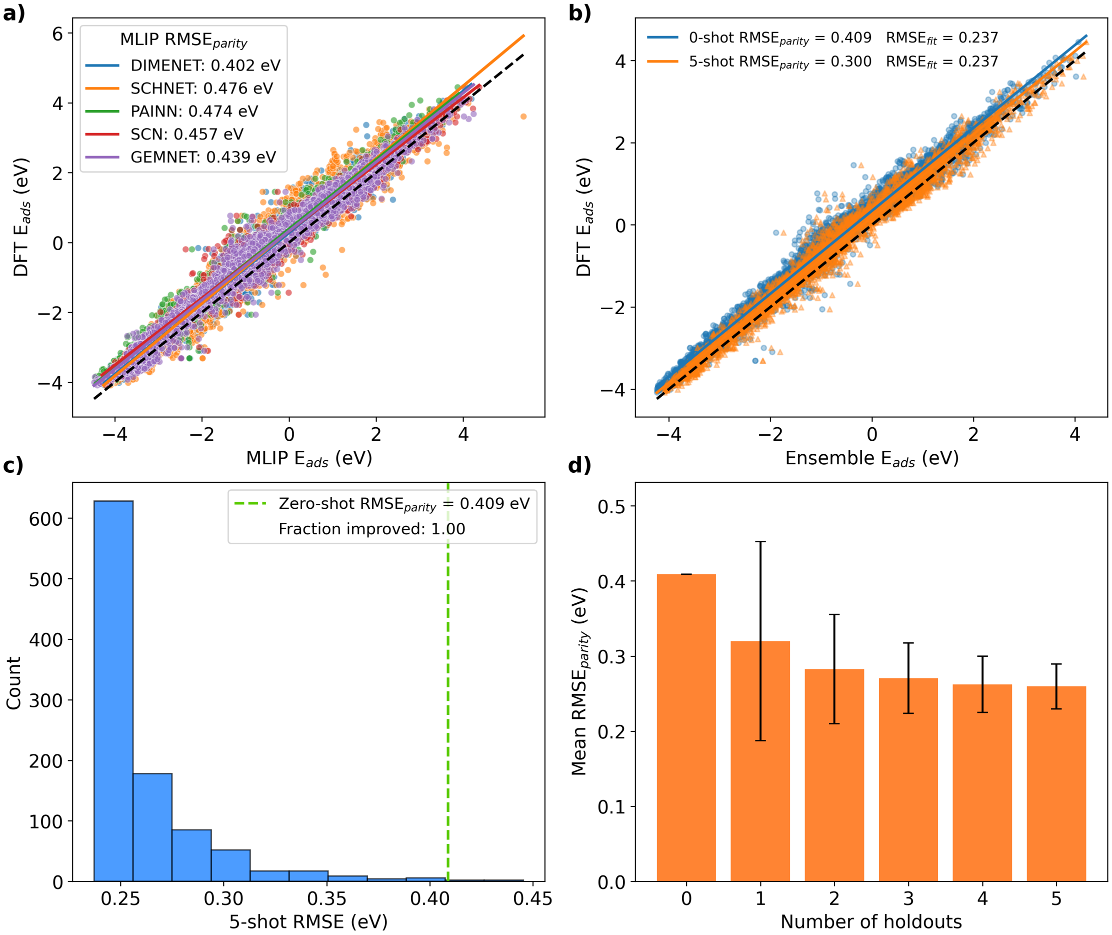

Figure S6: Few-shot performance on the O-BMA dataset after removing samples containing lanthanum.[1] The bias-correction behavior mirrors that observed for OH-BMA in the main text, including improved agreement with DFT, reduced ensemble RMSE, and systematic accuracy gains with increasing shot count.

Extended few-shot regime

To assess whether the bias-correction strategy continues to benefit from additional labeled references beyond those shown in the main text, we extended the few-shot expansion analysis up to ten shots on the OH-BMA and H-SAA datasets (Figure S7). While modest reductions in both the mean RMSE and its variance are observed as additional samples are incorporated, the improvements beyond five shots are marginal, indicating a saturation of performance in this regime. These results suggest that the dominant gains from bias correction are realized within the first few added references, motivating our focus on the 0- through 5-shot cases in the main text.

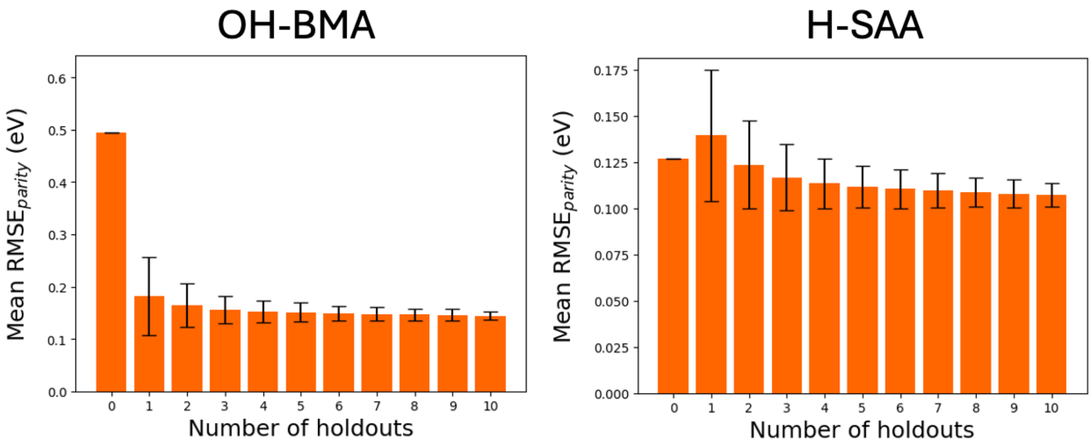

Figure S7: Mean RMSE, with standard deviations on the OH-BMA dataset[1] and the H-SAA dataset,[7] for 0 to 10 holdout samples. Mean RMSE improvements plateau around 5 samples.

We also observe that the sharpness uncertainty metric improves as additional samples are incorporated into the few-shot procedure (Figure S8), with gains saturating at approximately the same number of samples as the RMSE. This alignment suggests that sharpness improvement may serve as a practical stopping criterion for the few-shot method when focused on accuracy improvement.

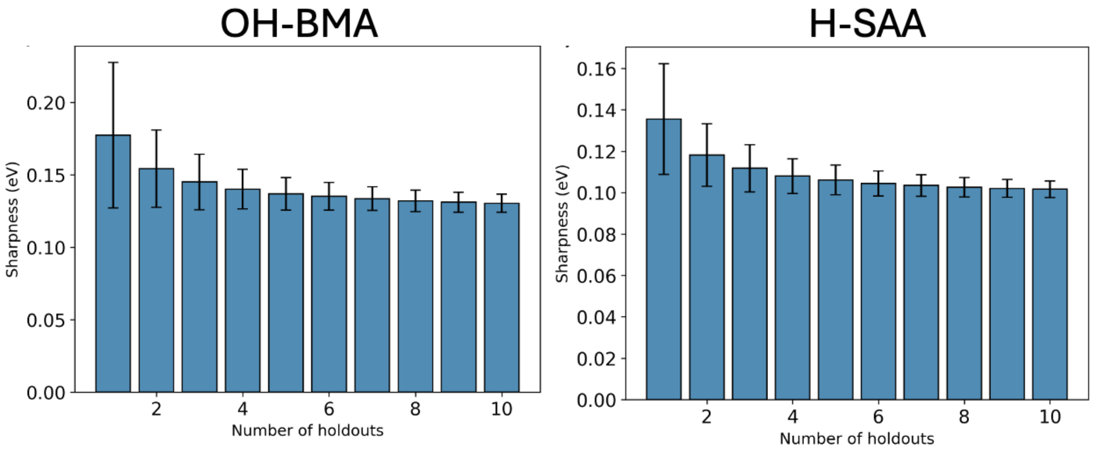

Figure S8: Mean sharpness, with standard deviations on the OH-BMA dataset[1] and the H-SAA dataset,[7] for 1 to 10 holdout samples. Mean sharpness plateaus around 5 samples.

MLIP subsets

To further probe the factors governing few-shot convergence on the H-SAA dataset, we analyzed the behavior of individual MLIPs and selected subsets of the ensemble. Figures S9 and S10 show that when MLIPs exhibit consistent overbinding or underbinding, either individually or after combining models with opposing biases, the few-shot RMSE decreases reliably once a sufficient number of correction samples is incorporated, and the overall adaptation rate remains similar despite differences in zero-shot performance. In contrast, Figure S11 illustrates that MLIPs with a mix of overbinding and underbinding, namely PAINN and SCN, exhibit slower early-stage convergence, consistent with reduced residual coherence. Together, these results indicate that few-shot adaptation depends primarily on the stability of the inferred bias rather than on ensemble composition or zero-shot accuracy alone.

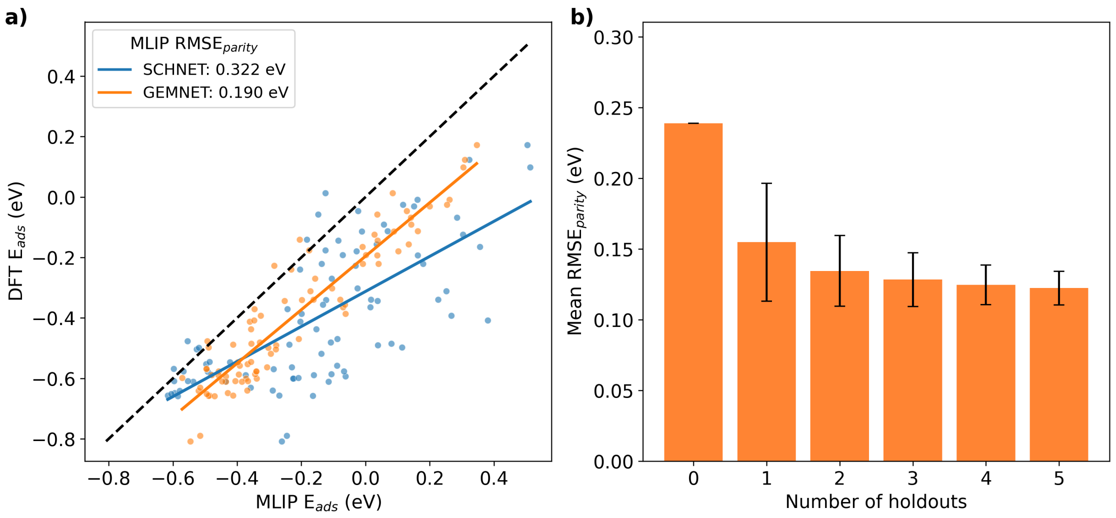

Figure S9: Few-shot adaptation for MLIPs that consistently underbind on the H-SAA dataset. **a)** DFT vs. SCHNet and GemNet adsorption energies. **b)** RMSE vs. number of holdouts. Because the MLIPs both underbind, the RMSE decreases rapidly as additional correction samples are incorporated.

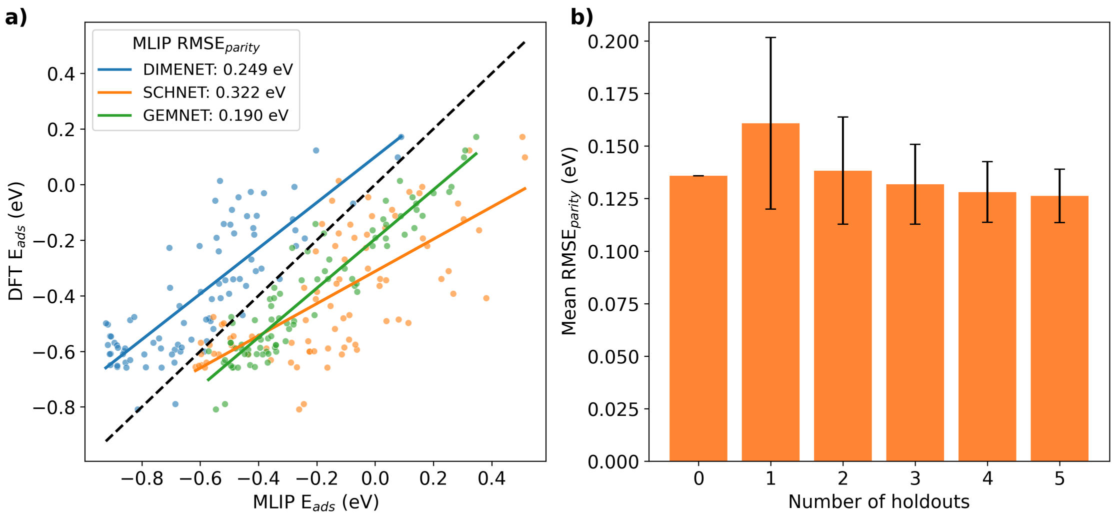

Figure S10: Few-shot adaptation when combining MLIPs with opposing directional biases on the H-SAA dataset. **a)** DFT vs. SCHNet, GemNet, and DimeNet adsorption energies. **b)** RMSE vs. number of holdouts. Because the MLIP errors partially cancel, zero-shot performance improves. The subsequent few-shot adaptation rate remains similar to that observed in Figure S9.

Post-hoc campaign analysis

Post-hoc analysis of the trimetallic search campaign (Figure S12) shows that bias correction adjusts the ensemble's overall energy scale, reflecting systematic bias removal rather than improvements in isolated predictions. Small calibration sets are sufficient to realign model

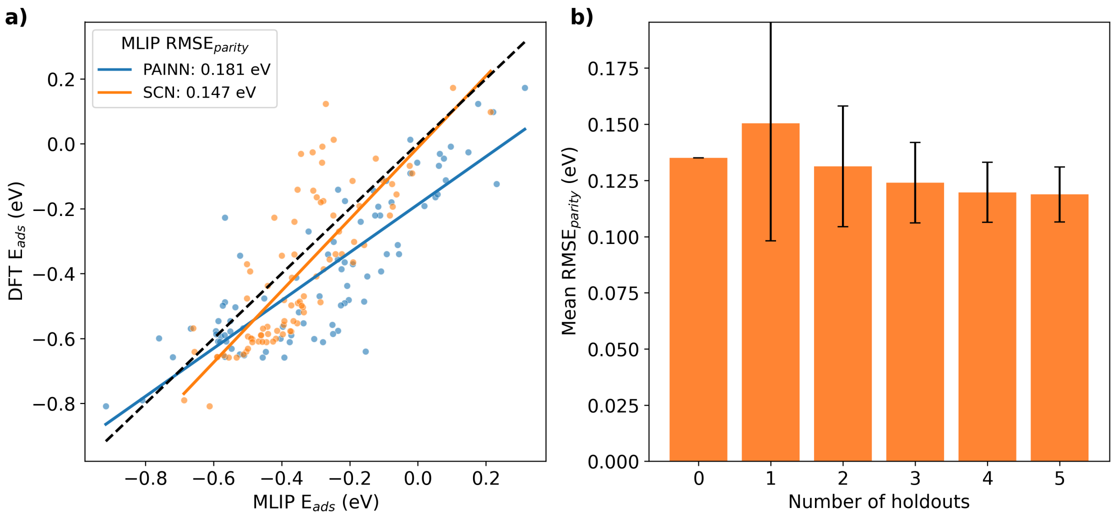

Figure S11: Impact of residual sign coherence on few-shot adaptation for the H-SAA dataset. **a)** PAINN and SCN exhibit substantial populations of predictions on both sides of the parity line, corresponding to mixed overbinding and underbinding behavior. **b)** This residual sign decoherence leads to conflicting early-stage bias corrections, resulting in slower convergence of the few-shot procedure.

predictions without retraining.

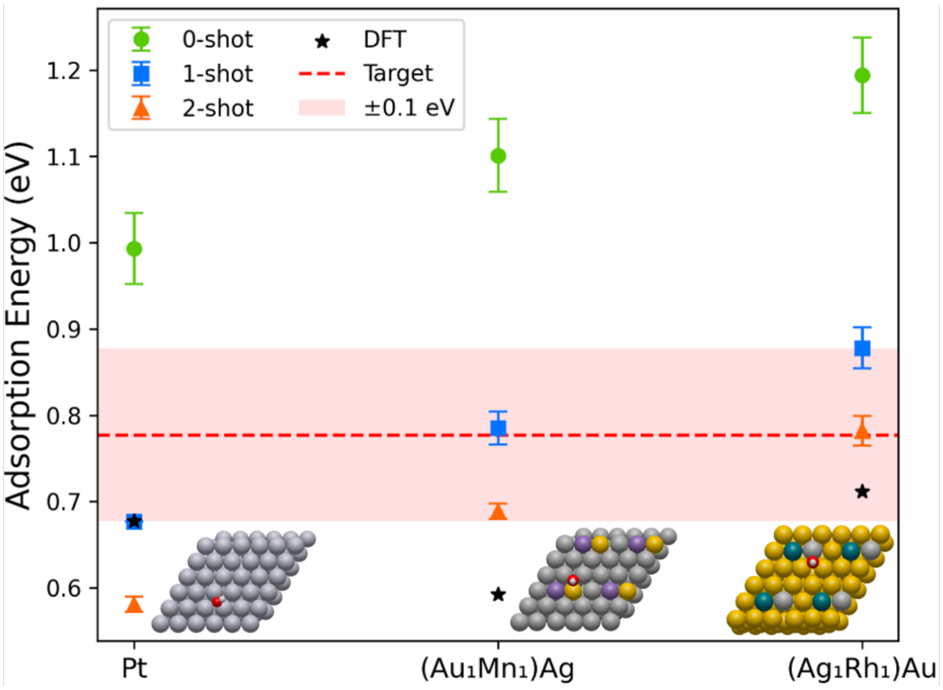

Figure S12: Screening campaign results, with every n-shot model applied to every surface for analysis. Bias correction steadily reduces systematic offsets between ensemble and DFT adsorption energies. Although the 1-shot correction gives a perfect match for Pt, this improvement reflects overfitting, whereas the 2-shot correction consistently improves predictions for the remaining surfaces.

References

(1) Mamun, O.; Winther, K. T.; Boes, J. R.; Bligaard, T. High-Throughput Calculations of Catalytic Properties of Bimetallic Alloy Surfaces. *Sci. Data* **2019**, *6*, 76, DOI: 10.1038/s41597-019-0080-z.

(2) Wu, J.; Yang, H. Platinum-Based Oxygen Reduction Electrocatalysts. *Acc. Chem. Res.* **2013**, *46*, 1848–1857, DOI: 10.1021/ar300359w.

(3) Darby, M. T.; Stamatakis, M. Single-Atom Alloys for the Electrochemical Oxygen Reduction Reaction. *ChemPhysChem* **2021**, *22*, 499–508, DOI: 10.1002/cphc.202000869.

(4) Viswanathan, V.; Hansen, H. A.; Rossmeisl, J.; Nørskov, J. K. Universality in Oxygen Reduction Electrocatalysis on Metal Surfaces. *ACS Catal.* **2012**, *2*, 1654–1660, DOI: 10.1021/cs300227s.

(5) Kim, H.; Yoo, T. Y.; Bootharaju, M. S.; Kim, J. H.; Chung, D. Y.; Hyeon, T. Noble Metal-Based Multimetallic Nanoparticles for Electrocatalytic Applications. *Adv. Sci.* **2021**, *9*, 2104054, DOI: 10.1002/advs.202104054.

(6) Gao, L.; Sun, T.; Chen, X.; Yang, Z.; Li, M.; Lai, W.; Zhang, W.; Yuan, Q.; Huang, H. Identifying the Distinct Roles of Dual Dopants in Stabilizing the Platinum-Nickel Nanowire Catalyst for Durable Fuel Cell. *Nat. Commun.* **2024**, *15*, 508, DOI: 10.1038/s41467-024-44788-0.

(7) Monasterial, A. P.; Hinderks, C. A.; Viriyavaree, S.; Montemore, M. M. When More Is Less: Nonmonotonic Trends in Adsorption on Clusters in Alloy Surfaces. *J. Chem. Phys.* **2020**, *153*, 111102, DOI: 10.1063/5.0022076.
